# Data-Independent Acquisition Phosphoproteomics of Urinary Extracellular Vesicles Enables Renal Cell Carcinoma Grade Differentiation

**DOI:** 10.1016/j.mcpro.2023.100536

**Published:** 2023-03-29

**Authors:** Marco Hadisurya, Zheng-Chi Lee, Zhuojun Luo, Guiyuan Zhang, Yajie Ding, Hao Zhang, Anton B. Iliuk, Roberto Pili, Ronald S. Boris, W. Andy Tao

**Affiliations:** 1 Department of Biochemistry, Purdue University, West Lafayette, Indiana, USA; 2 West Lafayette Junior/Senior Highschool, West Lafayette, Indiana, USA; 3 State Key Laboratory of Bioelectronics, School of Biological Science and Medical Engineering, Southeast University, Nanjing, Jiangsu, China; 4 Tymora Analytical Operations, West Lafayette, Indiana, USA; 5 Department of Medicine, Jacobs School of Medicine & Biomedical Sciences, University at Buffalo, Buffalo, New York, USA; 6 Department of Urology, Indiana University School of Medicine, Indianapolis, Indiana, USA; 7 Department of Chemistry, Purdue University, West Lafayette, Indiana, USA; 8 Department of Medicinal Chemistry and Molecular Pharmacology, Purdue University, West Lafayette, Indiana, USA; 9 Purdue Institute for Cancer Research, Purdue University, West Lafayette, Indiana, USA

## Abstract

Translating the research capability and knowledge in cancer signaling into clinical settings has been slow and ineffective. Recently, extracellular vesicles (EVs) have emerged as a promising source for developing disease phosphoprotein markers to monitor disease status. This study focuses on the development of a robust data-independent acquisition (DIA) using mass spectrometry to profile urinary EV phosphoproteomics for renal cell cancer (RCC) grades differentiation. We examined gas-phase fractionated library, direct DIA (library-free), forbidden zones, and several different windowing schemes. After the development of a DIA mass spectrometry method for EV phosphoproteomics, we applied the strategy to identify and quantify urinary EV phosphoproteomes from 57 individuals representing low-grade clear cell RCC, high-grade clear cell RCC, chronic kidney disease, and healthy control individuals. Urinary EVs were efficiently isolated by functional magnetic beads, and EV phosphopeptides were subsequently enriched by PolyMAC. We quantified 2584 unique phosphosites and observed that multiple prominent cancer-related pathways, such as ErbB signaling, renal cell carcinoma, and regulation of actin cytoskeleton, were only upregulated in high-grade clear cell RCC. These results show that EV phosphoproteome analysis utilizing our optimized procedure of EV isolation, phosphopeptide enrichment, and DIA method provides a powerful tool for future clinical applications.

## Highlights

• Optimal gas-phase fractionated (GPF) library for urinary EV phosphoproteomics.
• Integrating EVtrap, PolyMAC, and GPF DIA for thousands of unique EV phosphosite identification.
• Linear discriminant analysis (LDA) correctly clustered the grades of RCC patients.
• Chronic kidney disease (CKD) served as a better control for RCC biomarker screening.

## Graphical Abstract

**Figure:**
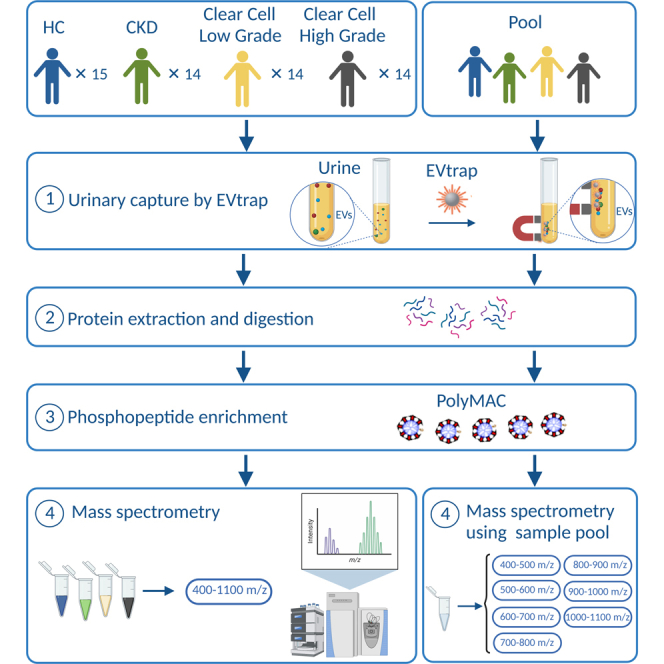

## In Brief

Considering the limitations of current approaches, it is necessary to develop a novel diagnostic technique for early intervention of renal cell carcinoma (RCC). Here, we made our efforts to adapt the existing DIA method to analyze urinary EV phosphoproteomics for noninvasive RCC biomarker screening. Combined with our in-house EVtrap method and PolyMAC enrichment, we quantified 2584 unique phosphosites. We observed unique upregulated phosphosites and pathways differentiating healthy control (HC), chronic kidney disease (CKD), low-grade, and high-grade clear cell RCC.

Renal cell carcinoma (RCC) is currently the eighth leading cause of cancer death in the United States, affects nearly 300,000 individuals worldwide each year, and is responsible for more than 100,000 deaths annually (1, 2). RCC originates from the renal cortex or the renal epithelial cells and accounts for more than 90% of all kidney cancers (3, 4). In the past decades, the incidence of RCC has been increasing steadily, and a diverse set of RCC subtypes has been recognized. The primary histologic subtypes are clear cell (70–80%), papillary (15%), chromophobe (5%), and unclassified RCC (5, 6). Distinct cytogenetic and immunohistochemical profiles characterize each subtype and prognoses as reflected by staging severity, with the lower stage being associated with longer survival rates (7). Clear cell RCC is the most common among these subtypes and accounts for the majority of RCC-related deaths. Due to the lack of symptoms until locally advanced or metastatic, renal cell cancer is typically detected incidentally when localized without warning. Currently, the detection and classification of renal masses rely on radiologic examinations, including ultrasound, computed tomography, magnetic resonance imaging, and so on. (8). In recent years, the frequent use of imaging for unrelated clinical symptoms of other diseases has led to a higher number of incidental diagnoses of RCC (9). Once identified the majority of renal masses are operated on without knowledge of subtype or grade. There are a number of explanations for this approach including a high number of historical nondiagnostic results, the risk of tumor seeding, and the risk of complications including primarily bleeding and pain as well as limited access to quality interventional radiology (10, 11, 12, 13, 14). Although recently renal mass biopsy has improved and utilization has increased, having an alternative office-based test that could predict tumor type, aggressiveness, and the need for surgical intervention while obviating the need for biopsy has been the elusive “holy grail” for the practicing urologist (8, 15). Considering that most of the identified tumors are low-grade, the alternative approach could allow the urologists to make decisions on a case-by-case basis depending on the grades of cancer, which might require active surveillance and different therapeutic strategies instead of surgical procedures.

**Fig. 1:**
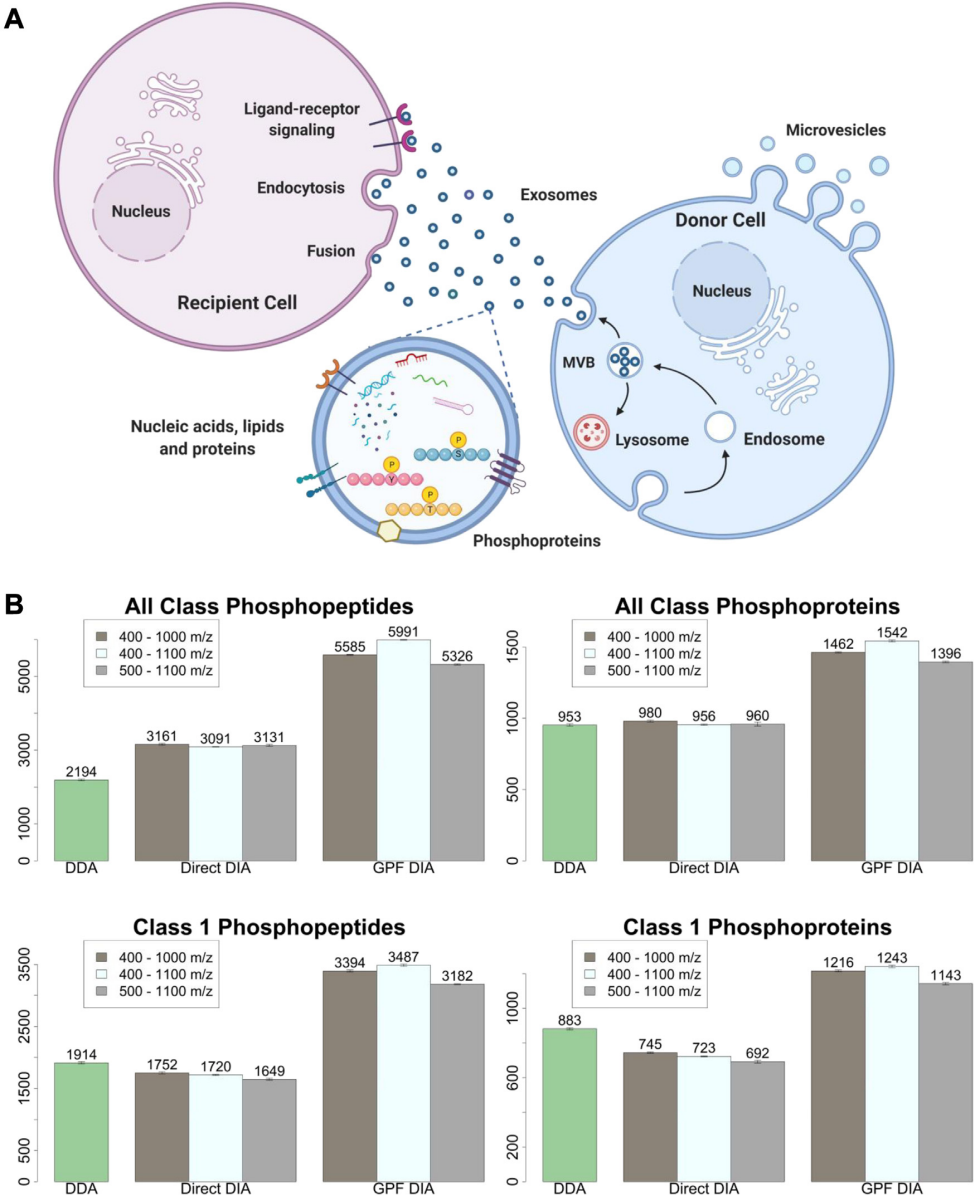
**DIA method optimization**. *A*, EVs, secreted from the parent cells, contain proteins, RNA, DNA, and lipids. Here, phosphoproteins directly reflect the cellular physiological status and signaling pathways during cancer progression. *B*, *top*: The number of all class phosphopeptides (*left*) and phosphoproteins (*right*) in DDA, direct DIA, and GPF DIA (with three different m/z ranges). *Bottom*: The number of class 1 phosphopeptides (*left*) and phosphoproteins (*right*) in DDA, direct DIA, and GPF DIA (with three different m/z ranges). Each experiment was performed in triplicates. Individual RAW files of the triplicates were searched separately. DDA, data-dependent acquisition; DIA, data-independent acquisition; EVs, extracellular vesicles; GPF, gas-phase fractionation; RCC, renal cell carcinoma.

Considering the limitations of current approaches, it is necessary to develop a novel diagnostic technique for early intervention of RCC. Therefore, early diagnosis and identification of RCC subtypes and tumor grades are essential to provide proper and effective treatment to increase the survival rate of patients. Recent studies suggest that extracellular vesicles (EVs) found in biofluids, such as urine, plasma, and saliva, can be a promising source for disease diagnosis (16, 17, 18). As shown in Figure 1*A*, EVs (*e.g.*, exosomes and microvesicles) are membrane-covered particles containing bioactive molecules such as RNA, DNA, proteins, and lipids secreted by all types of cells that are crucial for cell-to-cell communications (19, 20). Exosomes are nanoscale vesicles ranging from 30 to 120 nm with spherical or cup-like morphology, whereas microvesicles are irregular in shape and tend to be larger with a wide range of sizes up to approximately 1500 nm (21, 22). EVs secreted by cancer cells can promote cell growth and survival, shape the tumor microenvironment, and increase metastatic activities (23). Furthermore, EVs produced by cancer cells function as key mediators of cancer cell signaling and communication, causing adjacent or distant healthy cells to respond with phenotypic changes, which promote multiple aspects of tumor progression (23, 24). In addition, EVs are stably present in different body fluids, such as plasma and urine, which offer a useful and promising resource for non-invasive cancer biomarker discovery. In other words, EVs reflect the current disease state of cells by carrying bioactive components that can potentially be used as early biomarkers of RCC.

Phosphoproteins in EVs offer valuable surrogates for monitoring disease states, as phosphorylation is a key regulator of proteins involved in different cellular functions such as cell growth, differentiation, and apoptosis (25). Alterations in phosphorylation pathways are often associated with devastating diseases such as cancer (25). Some well-known signaling pathways, such as MAPK, EGFR/HER, CDK, and Cadherin–catenin complex, are major cell cycle players and deregulation of their phosphorylation–dephosphorylation activity has been shown to lead to the formation of various types of cancers. Previous studies from our group have identified numerous EV phosphoproteins as potential disease markers in urine and plasma for patients with breast cancer, chronic kidney disease, kidney cancer, and Parkinson’s disease (26, 27, 28).

A number of data-independent acquisition (DIA) methods have been introduced with increased coverage, sensitivity, and reproducibility compared to the more commonly used data-dependent acquisition (DDA) method and are powerful tools for disease biomarker discovery (29). Inspired by recent works by Searle *et al.* and Pino *et al.* (29, 30), we made our efforts to adapt the DIA method to analyze urinary EV phosphoproteomics. This study aimed to present an EV phosphoproteomics approach based on optimized DIA by comparing different DIA strategies for data acquisition methods, such as several windowing schemes and forbidden zones where no precursor could possibly exist (31), to profile phosphoprotein landscape in urinary EVs from patients with RCC and controls. A windowing scheme with windows edged by forbidden zones, designed to lessen the quadrupole transmission edge effects, maximizes the precursor ion transmission in the window range. As a result, there was a slight improvement in phosphopeptide identification. The windowing schemes were designed to be staggered, allowing peptide signal collection from multiple regions of the precursor space in the same MS2 and using the MS2 nearby in different precursor space regions to demultiplex signals specific to each region computationally. The demultiplexing during data processing separates the staggered precursor isolation windows into their effective parts, improves precursor selectivity by nearly a factor of 2, and eliminates potential noises (32).

In addition, we also compared two different strategies for DIA library building, including gas-phase fractionated (GPF) DIA library and direct DIA (an implementation of a library-free DIA method; Biognosys AG) (33). Searle *et al.* demonstrated that the GPF DIA library has the advantage of ensuring that the library is experiment-specific, always up-to-date, and accounts for variation across different instrument platforms without the need of doing offline fractionation for library generation (34). Moreover, the narrow window used by GPF-DIA provides parallel reaction monitoring quality data for every peptide inside the scanned range. On the other hand, direct DIA offers the benefit of performing DIA analysis without the need to build a library first. Taken together, we found that the 400 to 1100 m/z precursor range with GPF library was the most ideal for urinary EV phosphoprotein and phosphopeptide detection.

After we optimized the DIA phosphoproteomics method for urinary EV samples, we carried out DIA phosphoproteomics in urinary EVs derived from 57 individuals with low-grade clear cell RCC, high-grade clear cell RCC, chronic kidney disease (CKD), and healthy control (HC) to differentiate clear cell low-grade from CKD, controls, and clear cell high-grade RCC. We discovered that the GPF library provides the most comprehensive information for our clinical sample quantification. Some prominent cancer pathways, such as ErbB signaling, proteoglycans in cancer, renal cell carcinoma, and regulation of actin cytoskeleton, were found to be upregulated only in the high-grade clear cell RCC urinary EV samples. Furthermore, some phosphosites were uniquely upregulated in either clear cell low-grade or high-grade RCC. Several phosphosites known to be involved in RCC, such as PAK1 (T185) and BRAF (S365), were upregulated in clear cell high-grade. Moreover, the renal cell carcinoma pathway was only enhanced in clear cell high grade RCC. These findings offer opportunities for further exploration to develop novel EV phosphoprotein-based biomarkers and open the door for an effective early-stage clinical diagnosis for clear cell RCC.

## Experimental Procedures

### Sample Collection

Samples were collected at the Indiana University Simon Cancer Center and Methodist Research Institute in Indianapolis. The urine samples were collected on the same day right before the renal mass removal surgery. The collected urine samples were aliquoted into two or more cryotubes (5 ml) and stored in a −80 °C freezer. After the surgery, the obtained tumor tissue samples were used for immunohistochemistry analysis to diagnose the cancer subtypes and the grade based on the WHO/ISUP grading system for RCC (35). We have collected diverse RCC subtypes; however, we only utilized the low-grade and high-grade clear cell RCC for this study. Some samples were collected and used for the method optimization. We also collected urine samples from non-cancer healthy individuals (HC) and patients with chronic kidney disease (CKD). Here, we were also interested in investigating whether CKD samples could serve as a better control group than HC group when they were utilized as a control group for clear cell low-grade and clear cell high-grade RCC differentiation. In total, we processed 60 samples (15 HC, 15 CKD, 15 clear cell low-grade, 15 clear cell high-grade) (See supplemental Data S1 for complete clinical characteristics) chosen randomly from a larger sample cohort. All samples were collected under IRB approved protocol no. 1011004282, Development of a Biorepository for IU Health Enterprise Clinical Research Operations from Indiana Biobank. The study design and conduct complied with all relevant regulations regarding the use of human study participants and was conducted in accordance with the criteria set by the Declaration of Helsinki. All 60 samples were processed separately by implementing the statistical principles in experimental designs, including replication, randomization, and blocking when applicable (36).

### EV Isolation by EVtrap

The EVtrap beads were provided by Tymora Analytical Operations and were used as described previously (37). EVtrap, magnetic beads with functionalized lipophilic and hydrophilic groups, binds to the lipid bilayer membranes of EVs that enables fast and reproducible EV capture from urine samples. The 60 urine samples (approximately 10–15 ml each) and the sample pool (60 samples combined for a total volume of 180 ml) were centrifuged at 2500*g* for 10 min and frozen at −80 °C. After thawing, the sample pool was split into 6 × 50 ml tubes (30 ml in each tube) to facilitate the EVtrap incubation. Magnetic EVtrap beads were added to each urine sample at the ratio of 20 μl beads per 1 ml of urine. The samples, including the sample pool, were then incubated for 1 h by end-over-end rotation, allowing for ample movement of the beads. Afterward, the solution was removed with the aid of a magnetic separator. After three washes with PBS, the beads were incubated two times for 10 min by shaking with fresh 100 mM triethylamine to elute the EVs. After that, the eluted sample containing the EVs was collected, combined, and dried in a vacuum centrifuge.

### LC-MS Sample Preparation

The extracellular vesicles were solubilized with the help of a Phase Transfer Surfactant (PTS) buffer (38, 39). The PTS buffer included 0.5% (w/v)/12 mM sodium deoxycholate (SDC), 0.35% (w/v)/12 mM sodium lauroyl sarcosinate, and 50 mM Tris-Cl, 10 mM tris-(2-carboxyethyl)phosphine to assist reduction, 40 mM chloroacetamide to help alkylation, and phosphatase inhibitor to protect the phosphoproteins and phosphopeptides from phosphatases. 100 μl of PTS was added to each sample. The samples were boiled for 10 min at 95 °C in the dark and were diluted fivefold using 50 mM TEAB for digestion. Then, a BCA assay was performed to identify the amounts of proteins per sample. Lys-C was added at the fixed 1:50 w/w ratio, and samples were digested at 37 °C for 3 h in a shaker at 1100 rpm. Afterward, trypsin was added at the fixed ratio of 1 μg per 50 μg protein per sample, and samples were digested overnight at 37 °C.

Each sample was acidified with 50 μl of 10% trifluoroacetic acid (TFA) solution to adjust its final concentration to 1% TFA. 650 μl of ethyl acetate was added to the sample and vortexed for 2 min to precipitate the detergents completely. The samples were then centrifuged at 20,000*g* for 3 min to differentiate the aqueous and organic phases. The upper layer (organic phase) was removed, and the samples were dried for about 1.5 h until less than 150 μl remained. Another 1 ml of ethyl acetate was added to the samples, and the previous steps of vortexing and centrifugation were repeated. This time, the samples were dried completely.

TopTip C-18 (10–200 μl) (Glygen, Part number: TT2C18.96) tips were used to desalt the samples according to the manufacturer’s instructions. Protein concentration was checked using a Pierce Colorimetry peptide concentration assay kit, normalized for the same amount of peptides for each sample, and dried completely. The sample pool utilized 50 mg Sep-Pak columns (Waters) for desalting according to the manufacturer’s instructions. After the concentration examinations using the Pierce Colorimetry peptide concentration assay, the appropriate peptide concentration for the GPF library (seven injections) was placed into several tubes (for PolyMAC preparation) and dried completely.

Each sample, including the pooled samples, was subjected to phosphopeptide enrichment using the PolyMAC Phosphopeptide Enrichment kit (Tymora Analytical) according to the manufacturer's instructions (40). The eluted phosphopeptides were dried completely in a vacuum centrifuge.

### LC/MS-MS Analysis

The phosphoproteomic samples were spiked with an 11-peptide indexed Retention Time internal-standard mixture (Biognosys) to normalize the LC-MS signal between the samples. All samples were captured on a 2-cm Acclaim PepMap trap column and separated on a heated 50-cm Acclaim PepMap column (Thermo Fisher Scientific) containing C18 resin. The mobile phase buffer consisted of 0.1% formic acid in HPLC grade water (buffer A) with an eluting buffer containing 0.1% formic acid in 80% (vol/vol) acetonitrile (buffer B) run with a linear 85-min gradient of 5 to 35% buffer B at a flow rate of 300 nl/min. The UHPLC was coupled online with a Q-Exactive HF-X mass spectrometer (Thermo Fisher Scientific, see supplemental Data S2 for instrument settings). For DDA experiments, the mass spectrometer was run in the data-dependent mode, in which a full-scan MS (from m/z 375–1500 with the resolution of 60,000) was followed by MS/MS of the 15 most intense ions (30,000 resolution; normalized collision energy - 28%; automatic gain control target (AGC) - 2E4, maximum injection time - 200 ms; 60s exclusion). For DIA experiments, the mass spectrometer was run in the data-independent mode, in which a full-scan MS (polarity - positive; scan range 389.8–1109.8 m/z with the resolution of 60,000; automatic gain control target (AGC) - 1E6, maximum injection time - 60 ms; spectrum data type - centroid) was followed by MS/MS with 8.0 m/z staggered-isolation windows schemes as described in Searle *et al.* and Pino *et al.* (polarity - positive; 15,000 resolution; normalized collision energy - 27%; AGC - 1E6, maximum injection time - 20 ms; loop count - 88; spectrum data type - centroid) (29, 30).

### Construction of GPF and Direct DIA Library

The GPF spectra library was generated using Spectronaut Pulsar search (Biognosys, v15, Switzerland) according to Searle *et al.* and Pino *et al.* (29, 30). In brief, we acquired the GPF spectra library (n = 7) from narrow mass ranges: 394.8 to 504.8, 494.8 to 604.8, 594.8 to 704.8, 694.8 to 804.8, 794.8 to 904.8, 894.8 to 1004.8 m/z, and 994.8 to 1104.8 m/z at the MS1 level (See supplemental Data S2 for windowing schemes). For the MS2 level, 4 m/z DIA spectra were acquired for each MS1 range. The effective isolation window is only 2 m/z after deconvolution. All the DIA data were first searched against the human FASTA file from UniProt Swiss-Prot downloaded December 20, 2021 (78,120 total entries) with a default setting. Maximum of two missed cleavages were allowed for trypsin digested peptides with variable modification as acetylation on protein N terminus (+42.016 Da) and oxidation of methionine (+15.995 Da) residues as well as phosphorylation (+79.966 Da) on Ser, Thr, and Tyr residues. Fixed modification of carbamidomethylation (+57.022 Da) of cysteine residues was included. The tolerances for both MS1 and MS2 levels were set to dynamic (determined by Spectronaut based on the extensive mass calibration) with a correction factor of 1 (no correction). A cutoff of 1% FDR was set at PSM, peptide, and protein levels with an enabled phosphosite localization filter at 0.75. Fragment ions minimum m/z 300, maximum m/z 1800, the minimal relative intensity of 5%, and 15 most intense fragment ions per precursor were included, and those with less than three amino acid residues were not considered. Fragment ions with neutral losses were included. The empirical indexed Retention Time (iRT) database was used for the iRT reference strategy with deep learning-assisted iRT regression as a backup. Precursors with phosphorylation modification as well as non-modified were retained. The library search for the direct DIA was established using the same parameters as the GPF spectral library described above (33).

### DIA Method Optimization

The placing of the window edges at the forbidden zones for phosphorylation PTM was performed and compared to without the forbidden zones using a precursor range of 400 to 1000 m/z. The “forbidden zones” were adapted based on Pino *et al* (29). In brief, we used the mentioned formula in Pino *et al*:

$$ceil\;\left(\frac{nominal\;mass}{optimal\;\frac{m}{z}\;increment}\right)\times optimal\;\frac{m}{z}\;increment+optimal\;\frac{m}{z}\;constant$$

where the optimal m/z increment is 1.00045475 and constant is 0.18 (phospho-enrichment shift).

Then, we compared two different methods for DIA library generation: the GPF and direct DIA methods. Finally, three different precursor ranges (400–1000, 500–1100, and 400–1100 m/z) were compared.

### Experimental Design and Statistical Rationale

During DIA method optimization, we focused on interpreting results from DDA and different DIA acquisition strategies, rather than any biological interpretation. As such, triplicates of urine phospho-samples were injected to the mass spec with DDA and different DIA acquisition strategies (one MS run for each acquisition strategy). The different acquisition strategies were compared in terms of their phosphopeptides identification. To develop a DIA-based analytical strategy for clinical samples, specifically urinary EVs with RCC, we decided to only use 60 samples (15 HC, 15 CKD, 15 clear cell low-grade, and 15 clear cell high-grade biological replicates) by implementing the statistical principles in experimental designs, including replication, randomization, and blocking when applicable (36). Here, CKD was used as another control group. Before MS analysis, iRT Standard containing 11 artificial synthetic peptides was added to the phosphoproteomic samples. MS2 level quantitation was obtained. During the MS data analysis, three samples (one CKD, one clear cell low-grade, and one clear cell high-grade) were eliminated from further analysis, where one had deficient identifications and the other two did not meet normal distribution requirements. All abundances, which have normal distributions, were median-normalized for each sample, and *p*-value controlled Welch's two-sample *t* test was performed for each of the comparisons.

### DDA Data Analysis for Identification

The DDA raw file was searched directly against the human FASTA file from UniProt Swiss-Prot downloaded December 20, 2021 (78,120 total entries) with no redundant entries, using Sequest search engines in Proteome Discoverer 2.3 software (Thermo Fisher Scientific). The mass tolerance was set at 10 ppm for MS1 and 20 ppm for MS2. In the processing workflow, search criteria for the search engine were performed with full trypsin/P digestion, a maximum of two missed cleavages allowed on the peptides analyzed from the sequence database, carbamidomethylation on cysteines (+57.0214 Da) as a static modification, and oxidation (+15.9949 Da) on methionine residues, acetylation (+42.011 Da) at N terminus of proteins, and phosphorylation (+79.996 Da) on serine, threonine, or tyrosine residues as variable modifications. The false discovery rates of proteins and peptides were set at 0.01. All protein and peptide identifications were grouped, and any redundant entries were removed. The Phosphosites localization tool was applied to filter class 1 localization (class 1 probability cutoff ≥0.75). The total identification numbers of unique phosphopeptides and unique master phosphoproteins were reported.

### DIA Data Analysis for Both Identification and Quantification

The signal extraction and quantitation of the DIA data were performed in Spectronaut (Biognosys, v15), utilizing a standard setting with some modifications. RAW data from different acquisition methods were analyzed separately in different Spectronaut sessions. For CV calculation and identified feature overlaps, the “match between runs” feature in Spectronaut was used. Briefly, dynamic retention time prediction with local regression calibration was used. MS1 and MS2 interference corrections were enabled. The FDR at peptide precursor was set to 1%. Meanwhile, the protein level was analyzed with scrambled decoy generation and dynamic size at 0.1 fractions of library size. The quantitation was performed at the MS2 level by enabling local cross-run normalization. Qvalue percentile with 0.2 fraction and no imputing was used as data filtering. Phosphopeptide precursor abundances were measured by the sum of fragment ion peak areas, and peptide grouping was performed based on modified peptides. Phosphosites localization tool was applied to filter class 1 localization (class 1 probability cutoff ≥0.75 for libDIA, and ≥0.99 for direct DIA). It is recommended to apply a higher site confidence score cutoff of 0.99 to achieve error rates comparable to library-based DIA and DDA (33). For site-specific quantitation, the abundance of a phosphorylation site was calculated by summing up all of the abundances in precursors/peptides, which contain the site in the corrected precursor/peptide abundance file. Customized R scripts adapted from Kitata *et al.* (41) performed site-specific quantification.

### Statistical Analysis and Pathway Annotation

Further statistical analysis was performed by Perseus software (1.6.15.0) for the 60 urine samples (42). Those values with raw intensities below 30 were converted to NaN for quality control. All the phosphosite abundances were log_2_ transformed. The abundances were grouped into four different groups: HC, CKD, clear cell low-grade, and clear cell high-grade. Then, three samples (one CKD, one clear cell low-grade, and one clear cell high-grade) were eliminated from further analysis because they did not satisfy the abundance normal distribution requirement. The other 57 samples were further split into five comparisons: CKD vs. HC, clear cell low-grade vs. HC, clear cell high-grade vs. HC, clear cell low-grade vs. CKD, and clear cell high-grade vs. CKD. The phosphosites with more than 70% quantified abundances in at least one category were kept for each comparison. Imputation for the missing abundances was performed by assigning small values from the normal distribution (1.8 SDs downshift and 0.3 SDs width). Then, all abundances were further median-normalized for each sample. *p*-value controlled Welch's two-sample *t* test was performed for each of the comparisons (with cutoff values of *p*-value = 0.05 and log base 2 difference = 0.5, which equals ∼1.414 fold-change). Feature selection was done in R using the ranger package (43) and visualized using vip package (44). The multiclass ROC analyses were created using the pROC package (45). Protein–protein interaction network analysis and functional annotation were done by STRING database (version 11.0 b) (46) and Ingenuity Pathway Analysis (47). For this analysis, pathways from the biological processes, molecular functions, and Kyoto Encyclopedia of Genes and Genomes (KEGG) (48) databases were considered.

## Results

### Exploration of DIA-MS Strategies

The reproducibility and sensitivity of DDA MS is highly dependent on sample complicity and instrument conditions, and therefore, it is often less suitable for biomarker discovery research. To overcome DDA limitations, DIA has been developed that sliced the peptide ion space into segments for MS2 measurement to counterbalance the complexity of biological samples. DIA has been shown to improve reproducibility, quantitative precision, and proteome coverage compared to label-free DDA. The overall goal of using DIA in this study was to increase the coverage of EV phosphoproteomes while enjoying good quantitative accuracy of typical DIA strategies. The balance between these two goals is critical for successful DIA experiments. To accomplish this for our study, we focused on several objectives: (a) maximize the precursor ion transmissions, (b) generate the most efficient DIA library, and (c) maximize the total precursor range of targeted phosphopeptides.

The placing of forbidden zones for particular post-translational modifications (PTMs), particularly phosphorylation, has been discussed previously, but it was never tested with actual phospho-samples (29). For the first objective, we placed the window edges at the forbidden zones for phosphorylation PTM and discovered that the window edge placing slightly increased the phosphopeptide identification (supplemental Fig. S1, see supplemental Table S1 and supplemental Data S3 for more details). The slight increase was possibly due to less pronounced quadrupole transmission edge effects. In addition, the class 1 peptide level coefficient of variations (%) among three replicates with forbidden zones were slightly smaller than those without forbidden zones (supplemental Fig. S2*A*, see supplemental Fig. S3*A* for class 1 phosphopeptide overlaps).

For the second objective, a library must be generated before analyzing the data to overcome the complexity of DIA data. Typically, libraries are built from fractionated DDA data at the cost of significant additional work, loss of sample, and instrument time. These obstacles, along with dependence on the quality of library, make DDA-based library generation less suitable for more dynamic phosphoproteome clinical samples, where phosphorylation level changes frequently due to multiple factors. For example, the disease progression, immune response, and environmental factors will all have big impact on the phosphorylation level. Recently, several alternative approaches have been developed to overcome those challenges by building DIA-only chromatogram libraries, such as the GPF and direct DIA methods (29, 30, 33). To our knowledge, there have not been published reports comparing the two methods for phosphoproteome data prior to this study. Lastly, when comparing different precursor ranges (400–1000, 500–1100, and 400–1100 m/z), we found that the 400 to 1100 m/z precursor range with GPF library was the most ideal for urinary EV phosphoprotein and phosphopeptide detection (Fig. 1*B*, see supplemental Table S2 for a table format). The peptide-level CVs among three replicates obtained with DIA were smaller than those of DDA (supplemental Fig. S2*B*, see supplemental Fig. S3, *B*–*D* for class 1 phosphopeptide overlaps). Moreover, the 400 to 1100 m/z precursor range have lower CVs compared to other precursor ranges in both direct and GPF DIA. Together, we find that by optimizing the total precursor ranges, setting the window edges at forbidden zones, and using the GPF library for a single shot mass spectrometry run in triplicates of an EV sample, Spectronaut identified 41.1% (direct DIA) and 173.1% (GPF DIA) more of all-class phosphopeptides compared to DDA analyzed by Sequest alone (Fig. 1*B*, see supplemental Table S2 for a table format). For class 1 phosphopeptides, we identified 82.2% (GPF-DIA) more phosphopeptides than with DDA. We also found that most all-class phosphoproteins were class 1 phosphoproteins.

### GPF-DIA Provides Comprehensive Identification and Quantitation Results

**Fig. 2:**
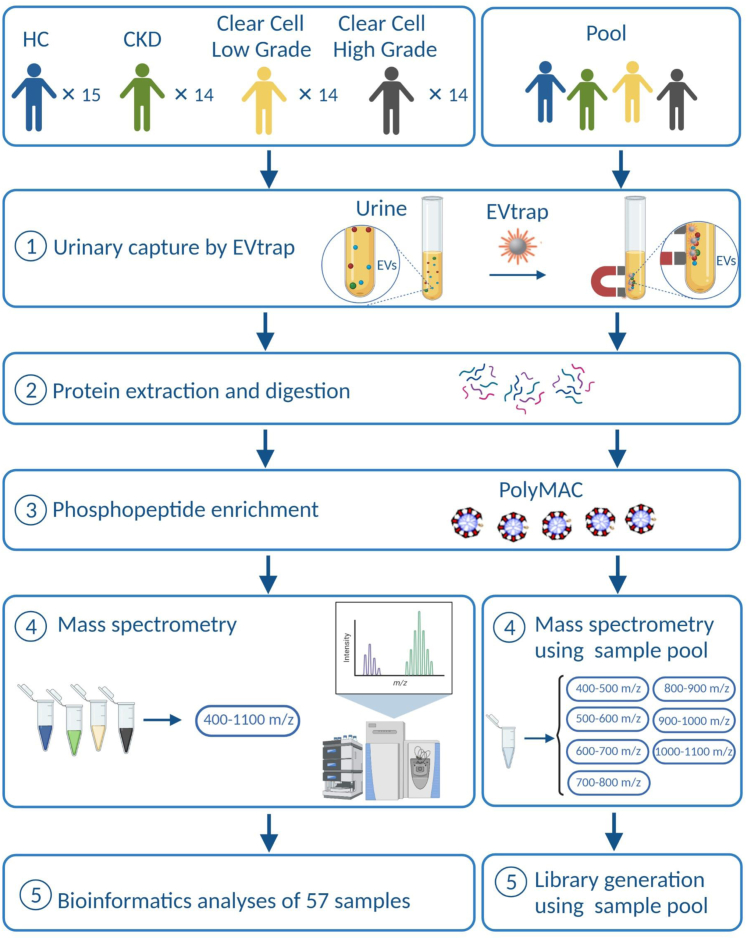
**Schematic diagram of the EV biological features and the application of the optimized method**. The workflow of the optimized DIA methods on 57 samples (where 3 other samples failed to fulfill the requirements for statistical analysis) representing 4 different groups, HC (healthy control), CKD (chronic kidney disease), Clear Cell Low-Grade RCC, and Clear Cell High-Grade RCC. The urine samples were processed using our in-house EVtrap for EV isolation and PolyMAC for phosphopeptides enrichment. DDA, data-dependent acquisition; DIA, data-independent acquisition; EVs, extracellular vesicles.

After finding the best DIA method for our MS data acquisition, we applied this optimized procedure to urine samples from 60 patients using approximately 10 to 15 ml urine from each (Fig. 2). Clear cell RCC is the most common RCC subtype, accounting for 75 to 80% of sporadic cases (49). The study's overall goal was to investigate whether our optimized method could be used to distinguish between low-grade and high-grade clear cell disease. In addition, we aimed to see whether CKD could be used as a better control group when investigating renal cell carcinoma biomarkers.

**Fig. 3:**
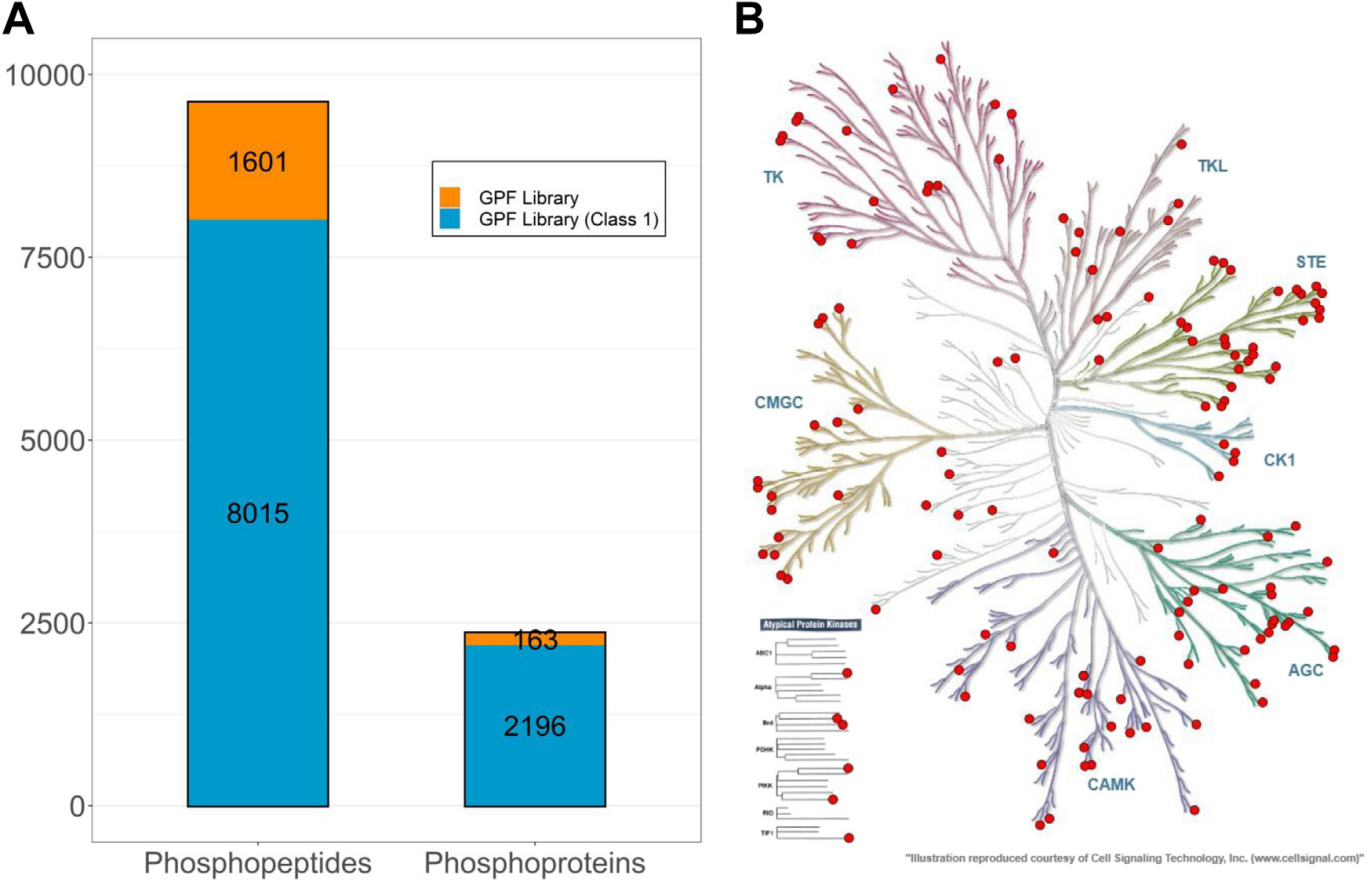
**Composition of the GPF library for the EV phosphoproteome system**. *A*, the number of phosphoproteins and phosphopeptides in the GPF library. *B*, kinase tree revealed 141 kinases in the GPF library. KinMap database (http://www.kinhub.org/kinmap/) was used with the input of protein accession number from GPF library. The kinase families listed include TK (tyrosine kinases), TKL (tyrosine kinase-like), CK1 (casein kinase 1), CAMK (calcium/calmodulin-dependent protein kinase), AGC (containing PKA, PKG, PKC families), CMGC (containing CDKs, MAPK, GSK, CLK families), and STE (serine/threonine kinases many involved in MAPK kinases cascade). EVs, extracellular vesicles; GPF, gas-phase fractionation.

High-quality and in-depth coverage spectral libraries are crucial for extensive DIA identification and quantitation. This is particularly important for EV samples because EV phosphoproteomics is only a small representation of total cell phosphoproteomics. In this case, we generated the GPF-DIA spectral library using the sample pool processed similarly to the individual samples. We identified 9616 all-class phosphopeptides and 2359 all-class phosphoprotein groups (8015 class 1 phosphopeptides and 2196 class 1 phosphoprotein groups) (Fig. 3*A*, see supplemental Data S4 for more details). The kinase tree revealed 141 kinases identified in the library (Fig. 3*B*). Throughout this study, the data analyzed by the GPF library were denoted GPF-DIA.

**Fig. 4:**
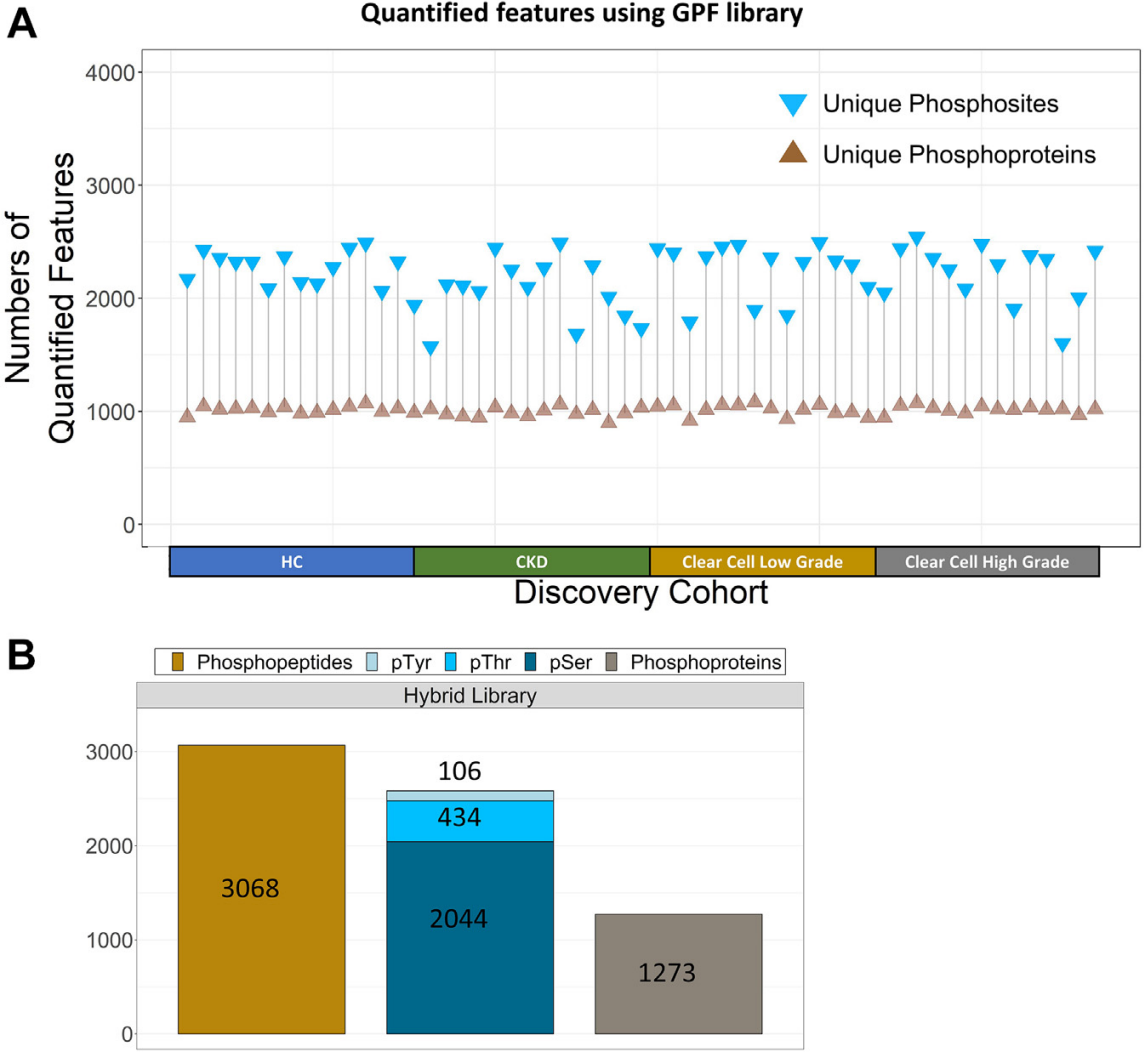
**The summary of identification and quantification for all 57 patients**. Cleveland Dot Plots for all quantified phosphoproteins and phosphosites searched using (*A*) GPF DIA across all 57 patients. *B*, the number of quantified phosphopeptides and phosphoproteins searched using GPF library along with the distribution of quantified serine (pSer), threonine (pThr), and tyrosine (pTyr) phosphosites. (All quantified features are class 1). DIA, data-independent acquisition; GPF, gas-phase fractionation.

The quantified class 1 features were visualized for every individual, where we could observe consistent numbers of phosphoproteins across 57 samples (Fig. 4*A*). Using the GPF-DIA method, our urinary EV phosphoproteomic analyses quantified 1273 unique phosphoproteins, 3068 unique phosphopeptide groups, and 2584 unique phosphosites (Fig. 4*B*, see supplemental Data S5 for more details). Regarding the phosphosite distribution in GPF-DIA, we quantified about 79% pSer, 17% pThr, and 4% pTyr (Fig. 4*B*, see supplemental Data S6 for more details). For our downstream analysis, we focused on those quantified 2584 phosphosites using the GPF-DIA (see supplemental Data S6) (42).

### The RCC Pathway is Only Upregulated in the High-Grade Clear Cell RCC Urine EVs

As described in the experimental procedure section, the quantified phosphosites must pass a rigorous statistical threshold and normalization criteria to be statistically useful. Out of 60, three samples failed to fulfill the requirements for statistical analysis, where one had deficient identifications and the other two did not meet normal distribution requirements. From a total of 57 samples, we then identified the upregulated phosphosites in CKD, low-grade and high-grade clear cell RCC groups against the healthy controls using volcano plots (with cutoff values of Welch's two-sample *t* test *p*-value = 0.05 and log base 2 difference = 0.5, which equals ∼1.414 fold-change, see supplemental Fig. S4*A*, see supplemental Data S7 for upregulated and downregulated phosphosites). We identified 300 upregulated phosphosites in CKD, 34 upregulated phosphosites in low-grade clear cell RCC, and 57 upregulated phosphosites in the high-grade clear cell RCC (supplemental Fig. S4*C*). Using those upregulated phosphosites, we performed feature selections to discover the top 15 from each comparison and visualized them in variable importance plots (supplemental Fig. S5*A*). The receiver–operating characteristic (ROC) analyses for the top seven phosphosites showed excellent area under the ROC curve (AUC) scores, which were more than 90% (supplemental Fig. S5*B*). Moreover, we discovered more upregulated phosphosites in the CKD vs. healthy control sample than in the other two comparisons. These upregulated phosphosites are associated with the CKD patients' immune responses (supplemental Fig. S6*A*).

**Fig. 5:**
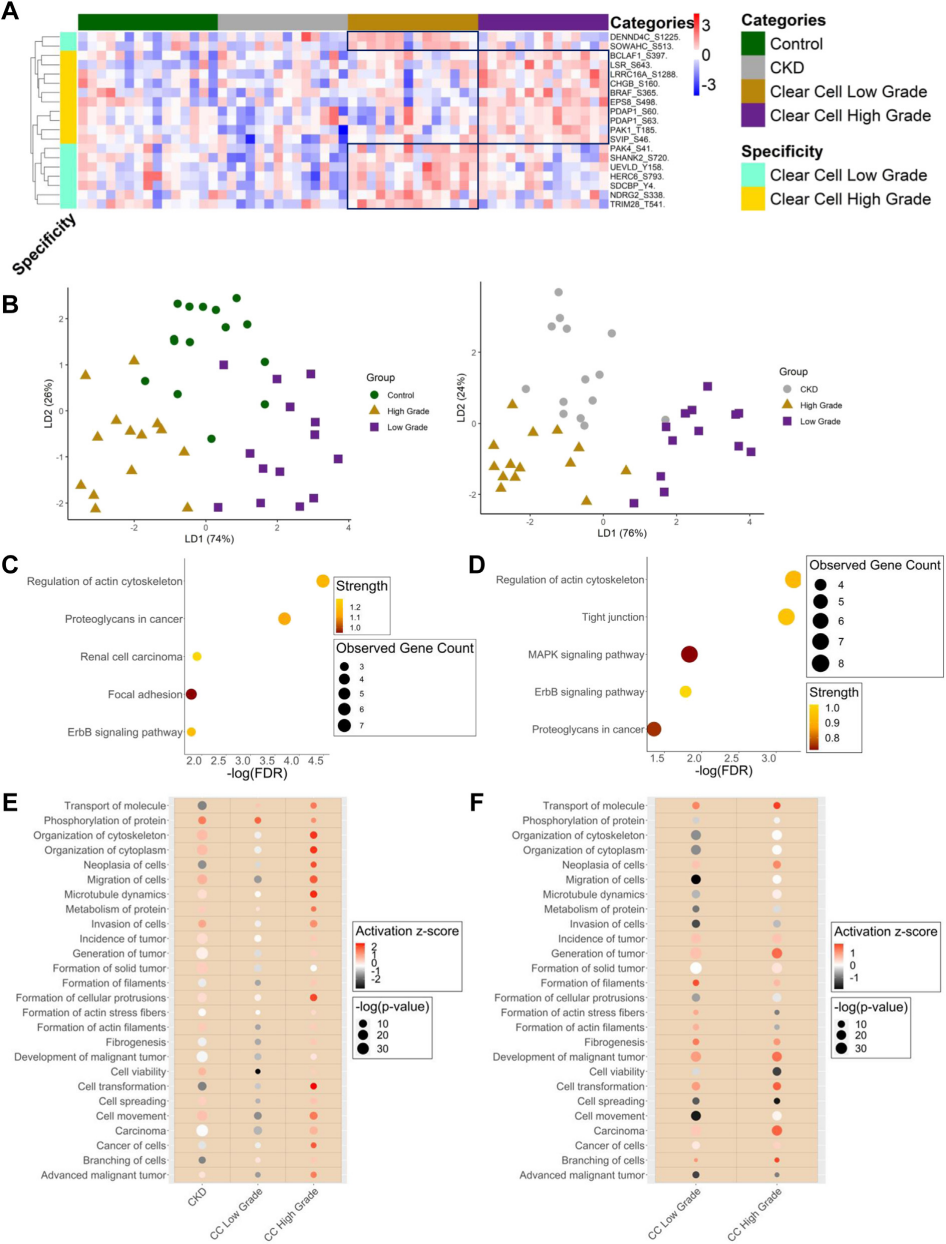
**The RCC subtype differentiation.***A*, the heatmap of upregulated phosphosites in clear cell low-grade and high-grade RCC. *B*, the linear discriminant analysis (LDA) plots for control, clear cell low-grade, and clear cell high-grade (*left*) and CKD, clear cell low-grade, and clear cell high-grade (*right*). *C*, the upregulated KEGG pathways in clear cell high-grade RCC *versus* control. *D*, the upregulated KEGG pathways in clear cell high-grade RCC *versus* CKD. *E*, diseases and functions analysis on CKD, clear cell low-grade, and clear cell high-grade RCC *versus* the control group (*left*). *F*, Diseases and functions analysis on clear cell low-grade and clear cell high-grade RCC *versus* the CKD group (*right*). CKD, chronic kidney disease; KEGG, Kyoto Encyclopedia of Genes and Genomes; RCC, renal cell carcinoma.

The most upregulated phosphosites in the low-grade and high-grade clear cell RCC samples are visualized in the heatmap in Figure 5*A* (*p*-value ≤0.1 when low-grade was compared to HC, CKD, or high-grade and when high-grade was compared to HC, CKD, or low-grade, calculated using the unpaired two-samples Wilcoxon test, see supplemental Fig. S7). PAK1, one of the class I p21-activated kinases, is a serine/threonine kinase involved in such cellular activities as neurogenesis, angiogenesis, cell migration, cytoskeletal dynamics, mitosis, apoptosis, and transformation (50). Highly phosphorylated PAK1 is associated with poor prognosis in patients with RCC (51, 52). Therefore, it is unsurprising that we found a phosphorylation site on PAK1 (T185) to be upregulated in a high-grade clear cell RCC (Fig. 5*A*). Interestingly, BRAF phosphorylation at S365 and S729 act as 14-3-3 protein binding sites and inhibit the activation of BRAF kinase activity (53). Furthermore, several phosphosites were found to be upregulated only in the low-grade clear cell RCC urinary EVs (Fig. 5*A*). Next, we asked whether CKD was a better control than healthy individuals in our data analysis. Using those upregulated phosphosites in Figure 5*A*, we performed supervised clustering linear discriminant analysis (LDA). The use of LDA for data classification has been widely used to classify many biological data sets such as cancer, HIV, and heart disease analysis (54). Here, when CKD was used as the control group, the CKD, low-grade, and high-grade patients were better clustered, outperforming the LDA when using the controls as the control group (Fig. 5*B*).

Gene ontology analysis was performed on the upregulated phosphosites in String proteins to obtain data on biological processes, molecular function, and the Kyoto Encyclopedia of Genes and Genomes (KEGG). In CKD, exocytosis, leukocyte activation, immune system process, and cell secretion were upregulated, mediating the immune system and inflammatory responses (supplemental Fig. S6*A*). This likely explains our results showing increased upregulated phosphosite identification in CKD compared to RCC samples. Meanwhile, the cytoskeleton organization-related pathways were found to be upregulated in the high-grade clear cell and are commonly dysregulated in cancer (supplemental Fig. S6*B*). Many high-grade clear cell molecular function pathways support the upregulation of cytoskeleton organization. For KEGG, the prominent cancer pathways, such as ErbB signaling, MAPK signaling, proteoglycans in cancer, RCC, and regulation of actin cytoskeleton, were found to be upregulated only in the high-grade clear cell RCC urinary EV samples (Fig. 5*C*). The renal cell carcinoma pathway included some already known phosphoproteins involved in RCC, such as PAK1, PAK2, and BRAF (51, 55, 56). These three phosphoproteins are also involved in ErbB and MAPK signaling pathways, focal adhesion, and regulation of actin cytoskeleton pathways.

We hypothesized that using the CKD as a control group could help eliminate the immune response from the more relevant cancer pathways analysis. We compared the upregulated phosphosites in clear cell RCC groups against the CKD cohort by using volcano plots (with cutoff values of Welch's two-sample *t* test *p*-value = 0.05 and log base 2 difference = 0.5, which equals ∼1.414 fold-change, see supplemental Fig. S4*B*, see supplemental Data S7 for upregulated and downregulated phosphosites). We identified 138 upregulated phosphosites in low-grade clear cell RCC and 129 upregulated phosphosites in the high-grade clear cell RCC samples (supplemental Fig. S4*D*). In these comparisons, the ROC analyses using the top 5 phosphosites also generated outstanding AUC scores (supplemental Fig. S5*B*). In this comparison, the endocytosis pathway was upregulated in the low-grade clear cell group (supplemental Fig. S6*C*). For KEGG of high-grade *versus* CKD, we observed similar pathways as previously shown when using the healthy controls as the control group. Most importantly, we observed significant upregulation of pathways relevant to the renal cell carcinoma pathway (Fig. 5*D*).

We also compared our upregulated pathways with a large proteogenomic study based on 103 clear cell RCC tissue samples (57). In that study, the authors created 30 phosphoproteomics co-expression network modules containing at least 20 genes in each and performed KEGG and Reactome (58) pathway enrichment analyses to identify biological pathways overrepresented in each network module *via* Fisher’s exact test. Although these phosphoproteomics clusters lacked differentiation between clear cell low-grade and high-grade RCC, these clusters displayed a general picture about what pathways were upregulated in clear cell RCC. Not surprising that those pathways discussed above, such as endocytosis, ErbB signaling, proteoglycans, RCC, and tight junction, were among those pathways enriched in the network module.

We further processed the data using the IPA, which was able to evaluate the results for both upregulated and downregulated phosphosites. First, we analyzed the data when the healthy individual samples were used as the control group. Here, we discovered a significant upregulation of protein phosphorylation in CKD (Fig. 5*E*) Many upregulated pathways in CKD were also upregulated in the high-grade clear cell RCC cohort. Interestingly, some of those upregulated pathways were related to cancer. Meanwhile, the advanced malignant tumor, carcinoma, cell transformation, and neoplasia of cell diseases and functions were upregulated in high-grade clear cell RCC alone (59).

Second, we used CKD as the control group, observing similar patterns as before (Fig. 5*F*). Those cancer diseases and functions, such as carcinoma, development of malignant tumor, generation of tumor, and neoplasia of cells were upregulated in the high-grade clear cell but not in the low-grade clear cell RCC. When CKD was used as the control group, the difference between activated diseases and functions in the low-grade clear cell RCC samples became much more distinct due to the less pronounced immune response related to phosphosites, rationalizing our experimental design to have patients with CKD as the control group as well.

## Discussion

Early diagnosis and monitoring of cancer through biofluids, such as blood or urine, has been a decades-long aim of medical diagnostics (60). Since protein phosphorylation is one of the most important and widespread molecular regulatory mechanisms that control almost all aspects of cellular functions, analysis of the phosphorylation network can conceivably provide clues regarding disease states (61). Traditionally, studying disease biomarkers from biofluids has been challenging due to the presence of phosphatases and high-abundant components in biofluids. However, the discovery of EVs opened new opportunities for developing phosphoproteins as potential cancer markers. Therefore, EVs have emerged as a rich resource for discovering tumor-relevant biomarkers from biofluids, such as urine, blood, and so on. (62). Particularly promising are the discoveries that these EV-based disease markers can be identified well before the onset of symptoms or physiological detection of a tumor, making them promising candidates for early-stage cancer detection and disease diagnosis (63). In addition, EVs are membrane-covered nanoparticles, protecting the inside contents from external proteases and other enzymes (64, 65, 66). This attribute makes them highly stable in a biofluid for extended periods of time, a highly valuable feature for clinical settings. These features also make EVs a promising source for developing phosphoproteins as disease markers, considering many phosphorylation events directly reflect cellular physiological status. Together, these EV advantages allow EV phosphoproteins to be used as a promising source for RCC biomarker discovery.

DIA has become a powerful quantitative mass spectrometry technique, allowing researchers to identify and quantify time-sensitive phosphoproteomes in clinical samples (41). To achieve our overall goal, we explored the DIA method to differentiate between low-grade and high-grade clear cell RCC. Here, we compared several DIA strategies to find the best suitable method for our mass spectrometry analysis to accomplish the objectives of our project. We found the 400 to 1100 m/z precursor range with forbidden zones, designed to lessen the quadrupole transmission edge effects, to be the most compatible method with our mass spectrometry system. When analyzing a single sample run in triplicates, we showed that GPF-DIA gave higher identification than DDA analyzed by Sequest and the direct DIA. The GPF-DIA fractionated and simplified the samples during the LC-MS data acquisition at the quadrupole; therefore, GPF-DIA provided a more in-depth library than direct DIA and increased the identification number for a single sample run, as visualized in Figure 1*B*.

In the main experiment (Fig. 2), we hypothesized that analyzing GPF-DIA phosphosites (2584 phosphosites depicted in Fig. 4*B*) would be valuable and would provide additional information about the cancer state. Indeed, we could find relevant phosphosites that were overexpressed exclusively in low-grade or high-grade clear cell RCC EV samples. We performed feature selections and ROC analyses and discovered that those top phosphosites have excellent AUC scores (supplemental Fig. S5). However, we need to emphasize that these analyses were performed on a limited number of sample sizes. Therefore, it is important to evaluate the model’s performance on a separate validation or test sets when they are available. Some of those phosphosites, visualized in Figure 5*A*, were already known to be involved in the clear cell RCC onset or progression. Those upregulated phosphosites in Figure 5*A* were able to differentiate the different categories into their corresponding groups using LDA as depicted in Figure 5*B*. Our collective phosphosite data supported by prior literature offers a great opportunity for further validation studies to translate these potential non-invasive signaling markers. Moreover, because we found that CKD could serve as better control group to differentiate low-grade and high-grade RCC (Fig. 5*B*), CKD offers the potential to be utilized as control group to study other types of RCC.

The gene ontology analysis of the unique upregulated phosphosites showed that renal cell carcinoma pathways were upregulated only in the high-grade clear cell RCC group. These pathways included some already known phosphoproteins involved in renal cell carcinomas, such as PAK1, PAK2, and BRAF (51, 55, 56). Along with the renal cell carcinoma pathway, ErbB and MAPK signaling, proteoglycans, and tight junction seen in Figure 5, *C* and *D*, were among those pathways enriched clear cell RCC as shown in the tissue-based study (57). Identifying many general cancer-related proteins in our data is expected, as many pathways are common in multiple cancer types. Indeed, in this study, we could identify pathways commonly observed in multiple cancer types but also a number of phosphoproteins that might be developed as RCC biomarkers.

Meanwhile, elevated phosphorylation on TRIM28, NDRG2, SDCBP, HERC6, UEVLD, SHANK2, PAK4, DENND4C, and SOWAHC was only discovered in low-grade clear cell RCC (Fig. 5*A*). The low-grade markers in Figure 5*A*, assessed with further validation, could help urologists determine the best treatments and convince those newly diagnosed patients with low-grade clear cell RCC to undergo active surveillance instead of going straight to surgeries that could result in potential overtreatments and overspendings.

In conclusion, we have developed a strategy to utilize phosphoproteins in urinary extracellular vesicles for RCC grade differentiation. The study highlights our ability to isolate and identify thousands of unique phosphosites utilizing the EVtrap method and PolyMAC enrichment in combination with GPF-DIA analyses. These findings further promote the underlying principle that this novel strategy could be valuable for exploring existing resources in any diseases. Finally, we expect that our results, followed by extensive validation with a larger cohort of clinical samples, could significantly augment the practitioner’s assessment of a localized renal mass through a simple office-based test and optimize management for many patients. Moreover, as a follow-up to this study, we have collected many additional samples with more diverse RCC subtypes and plan to apply the optimized method combined with an automation instrument for EV isolation to produce more reproducible results and validate those upregulated phosphosites uniquely found in low-grade or high-grade clear cell RCC. Overall, this project has created a translational environment where the collision of research with clinical perspectives has fostered the effective development of novel technologies and strategies to improve the effectiveness of cancer detection, diagnosis, and treatment.

## Data Availability

The mass spectrometry raw data files, Spectronaut, and Proteome Discoverer search results have been deposited in the MassIVE database (https://massive.ucsd.edu/ProteoSAFe/static/massive.jsp) and can be accessed *via* dataset identifier: MSV000091069 | PXD039504.

## Supplemental data

This article contains supplemental data.

## Conflict of interests

The authors declare a competing financial interest. A. I. and W. A. T. are principals at Tymora Analytical Operations, which developed EVtrap beads and commercialized PolyMAC enrichment kit.
