# Supplementary material for: Data-Independent Acquisition Phosphoproteomics of Urinary Extracellular Vesicles Enables Renal Cell Carcinoma Grade Differentiation
Source: Mol Cell Proteomics. 2023 Mar 29;22(5):100536. doi: 10.1016/j.mcpro.2023.100536 (PMC10165457; doi:10.1016/j.mcpro.2023.100536)
Supplement: Supplementary Figures [file mmc1.pdf]

Data-Independent Acquisition Phosphoproteomics of Urinary Extracellular Vesicles Enables Renal Cell Carcinoma Grade Differentiation

Marco Hadisurya[1]; Zheng-Chi Lee[1,2], Zhuojun Luo[1]; Guiyuan Zhang[3]; Yajie Ding[3]; Hao Zhang[3]; Anton B. Iliuk[4]; Roberto Pili[5]; Ronald S. Boris[6*]; and W. Andy Tao[1,4,7,8,9*]

[1]Department of Biochemistry, Purdue University, West Lafayette, IN 47906

[2]West Lafayette Junior/Senior Highschool, West Lafayette, IN 47906

[3]State Key Laboratory of Bioelectronics, School of Biological Science and Medical Engineering, Southeast University, Nanjing, Jiangsu, China

[4]Tymora Analytical Operations, West Lafayette, IN 47906

[5]Department of Medicine, Jacobs School of Medicine & Biomedical Sciences, University at Buffalo, Buffalo, NY 14203

[6]Department of Urology, Indiana University School of Medicine, Indianapolis, IN 46202

[7]Department of Chemistry, Purdue University, West Lafayette, IN 47907

[8]Department of Medicinal Chemistry and Molecular Pharmacology, Purdue University, West Lafayette, IN 47907

[9]Purdue Institute for Cancer Research, Purdue University, West Lafayette, IN 47907

[*]To whom correspondence should be addressed. Email: rboris@IUHealth.org; watao@purdue.edu

Supplementary Table 1

DIA Method	Proteome	Peptide	All Class Phosphoproteome	All Class Phosphopeptide
DIA without Forbidden Zone	751	2554	328	828
	809	2688	354	906
	819	2730	366	901
DIA with Forbidden Zone	848	2724	386	943
	809	2792	352	952
	763	2572	347	897

Supplementary Table 1. First DIA method optimization. A random urine EV sample was used to compare DIA without and with forbidden zones. Direct DIA was performed for each triplicate.

Supplementary Table 2

	Level	Localization Probability	Average of 3 Replicates		
DDA	Phosphoprotein	0	953.00		
		0.75	882.67		
	Phosphopeptide	0	2193.67		
		0.75	1914.00		
				GPF-DIA	Direct-DIA
	Level	m/z	LocProb	Average of 3 Replicates	Average of 3 Replicates
DIA	Phosphoprotein	400 - 1000 m/z	0	1462.33	980.33
			0.75	1216.33	744.67
		400 - 1100 m/z	0	1542.33	955.67
			0.75	1242.67	723.00
		500 - 1100 m/z	0	1396.00	959.67
			0.75	1142.67	692.33
	Phosphopeptide	400 - 1000 m/z	0	5585.00	3161.00
			0.75	3394.00	1752.00
		400 - 1100 m/z	0	5991.00	3090.67
			0.75	3487.33	1720.00
		500 - 1100 m/z	0	5326.00	3130.67
			0.75	3182.00	1649.33

Supplementary Table 2. Second DIA method optimization. A random urine EV sample was used to compare DDA, direct DIA, and GPF DIA with 0.75 and 0.00 minimal localization thresholds. The direct DIA and the GPF DIA were performed at three different precursor ranges: 400-1000, 500-1100, and 400-1100 m/z**.** Each experiment was performed in triplicates.

Supplementary Figure 1

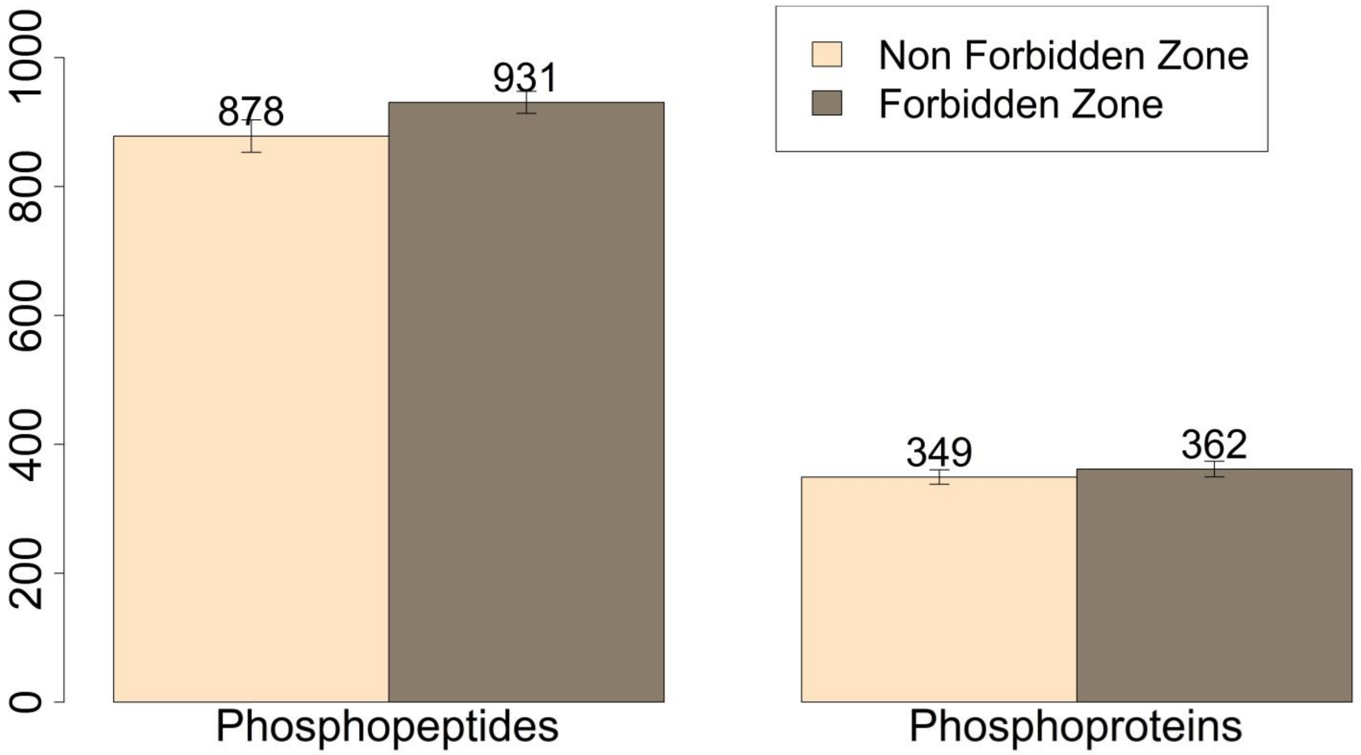

Supplementary Figure 1. First DIA method optimization. A random urine EV sample was used to compare DIA without and with forbidden zones. Direct DIA was performed for each triplicate. Individual RAW files of the triplicates were searched separately.

Supplementary Figure 2

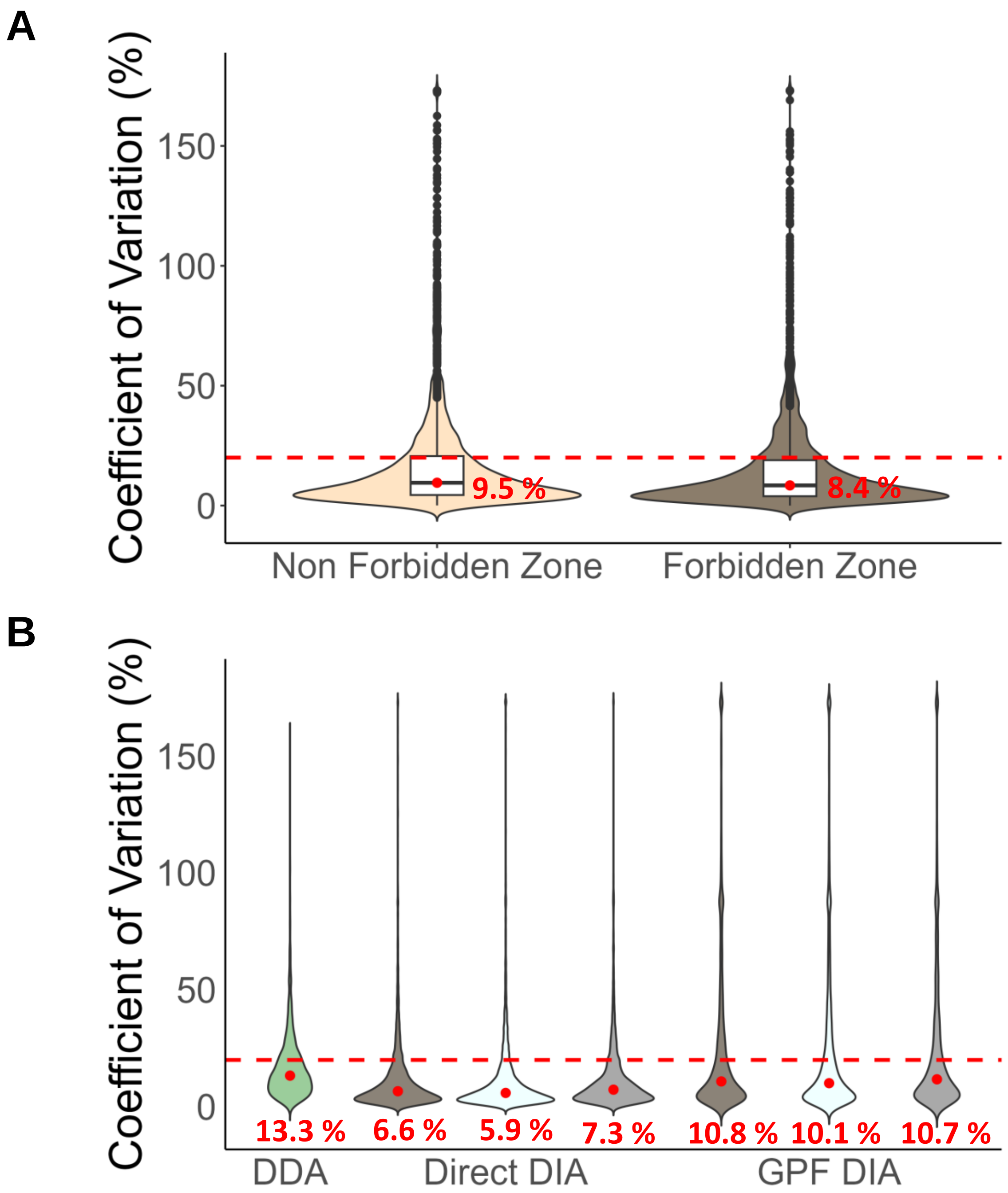

Supplementary Figure 2. The quantitative accuracy for class 1 phosphopeptides. A) The coefficient of variation (CV, %) of phosphopeptides obtained using DIA without and with forbidden zones. B) The CV (%) of phosphopeptides obtained using DDA, direct DIA, and GPF DIA (with three different m/z ranges). The CV (%) median is listed for each method. The red line indicates the CV at 20%. All quantified phosphopeptides were obtained using the “match between runs” of the triplicates in Spectronaut.

Supplementary Figure 3

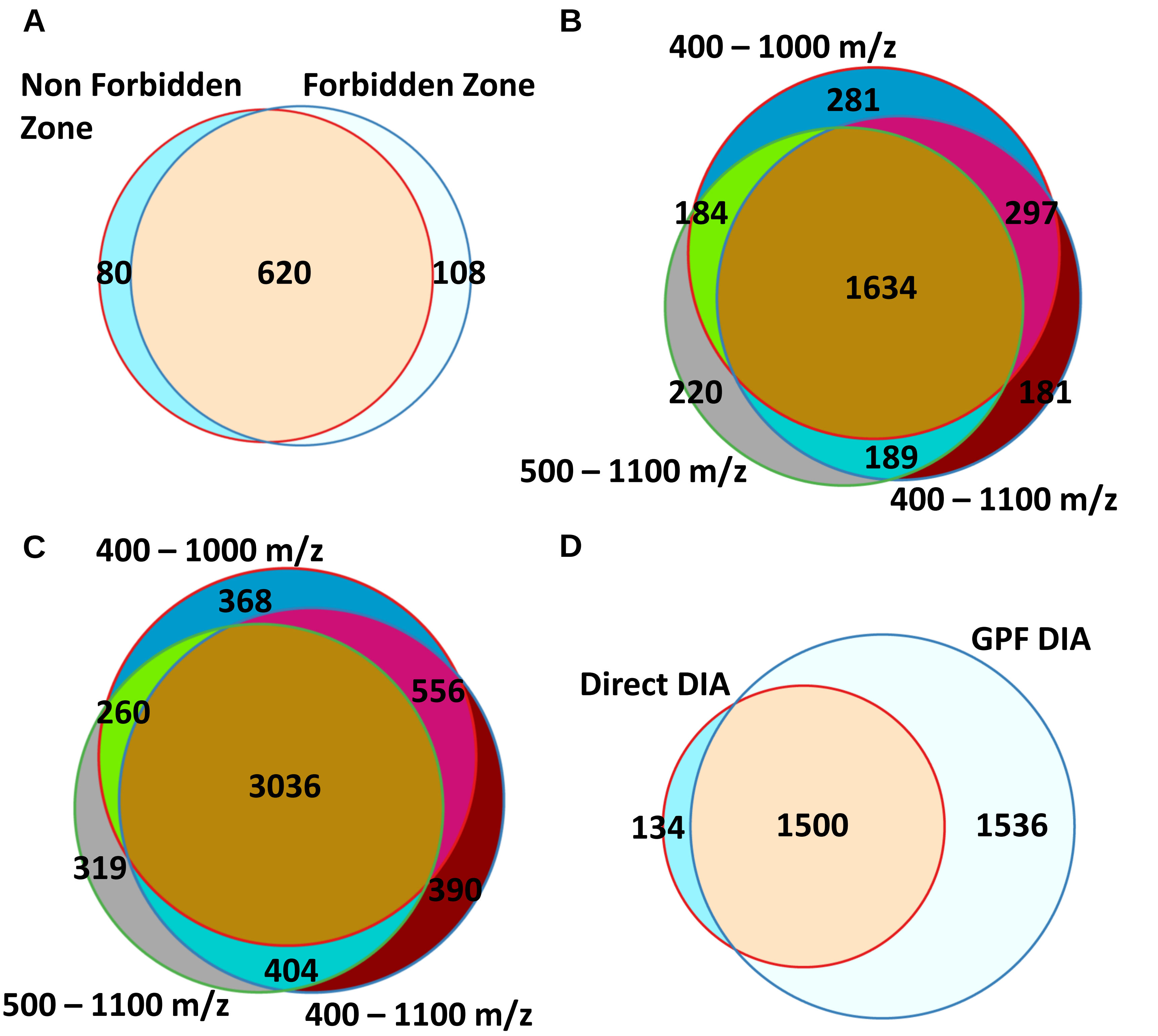

Supplementary Figure 3. The overlaps of class 1 phosphopeptides. The Venn diagram of phosphopeptides for (A) DIA without and with forbidden zones, (B) direct DIA with different precursor ranges, (C) GPF DIA with different precursor ranges, and (D) both direct DIA and GPF DIA (only common phosphopeptides from different precursor ranges colored in gold). All identified phosphopeptides were obtained using the “match between runs” of the triplicates in Spectronaut.

Supplementary Figure 4

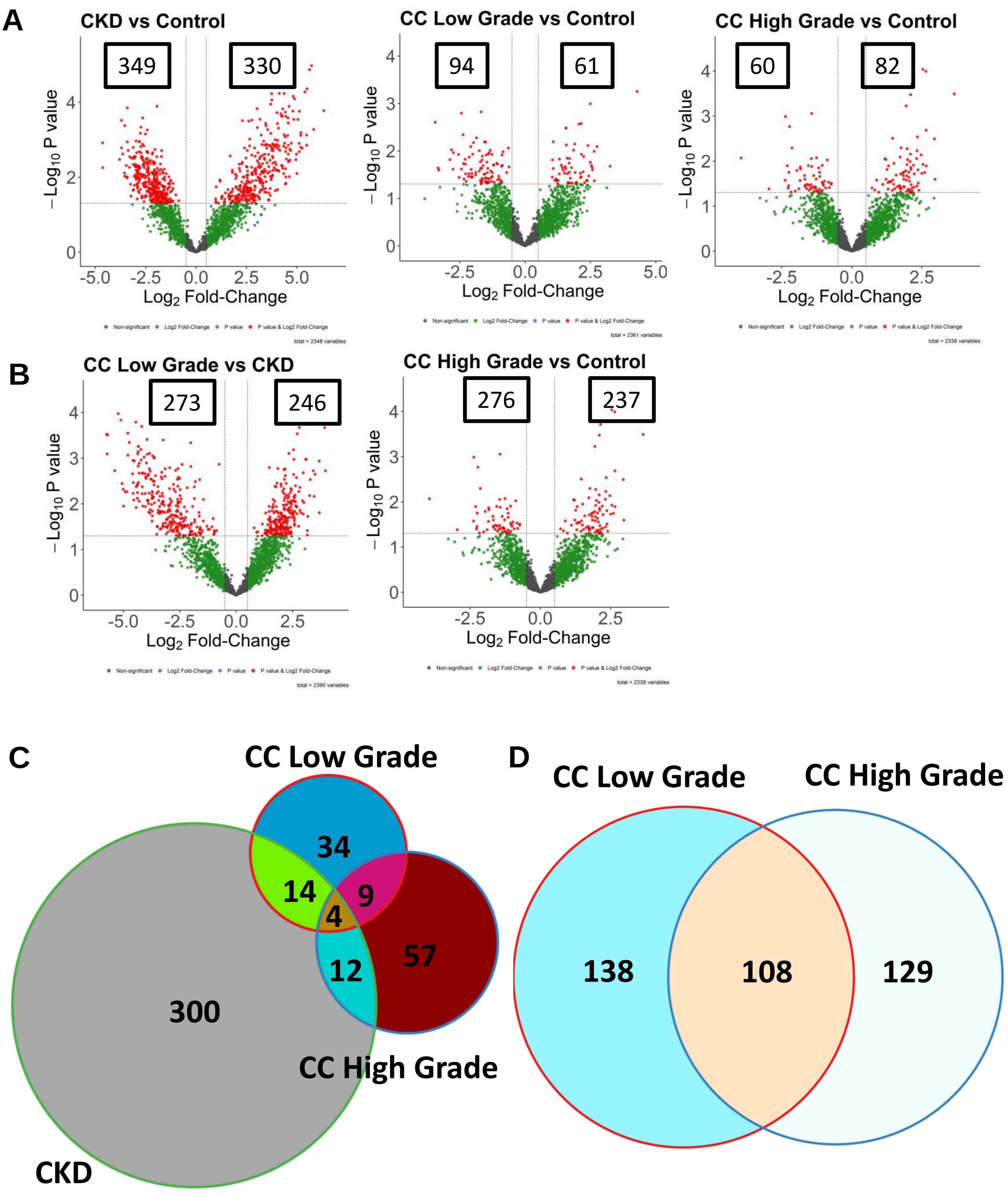

Supplementary Figure 4. Upregulated phosphosite identification. A) All three groups: CKD, clear cell low-grade, and clear cell high-grade were compared to the Control group. B) Two groups: clear cell low-grade and clear cell high-grade were compared to the CKD group. Significantly up-regulated phosphosites compared to the control (C) and compared to the CKD (D) were overlapped in Venn diagrams. Volcano plots were created for each comparison with cut-off values of Welch's two-sample t-test p-value = 0.05 and log base 2 difference = 0.5, which equals to ~1.414 fold-change.

Supplementary Figure 5

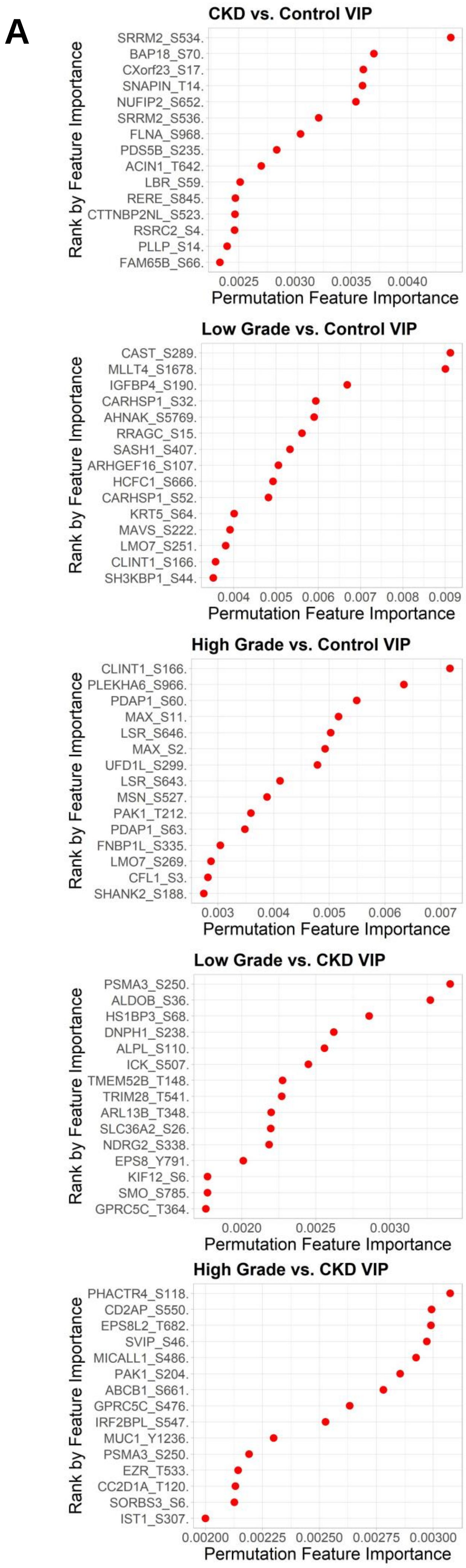

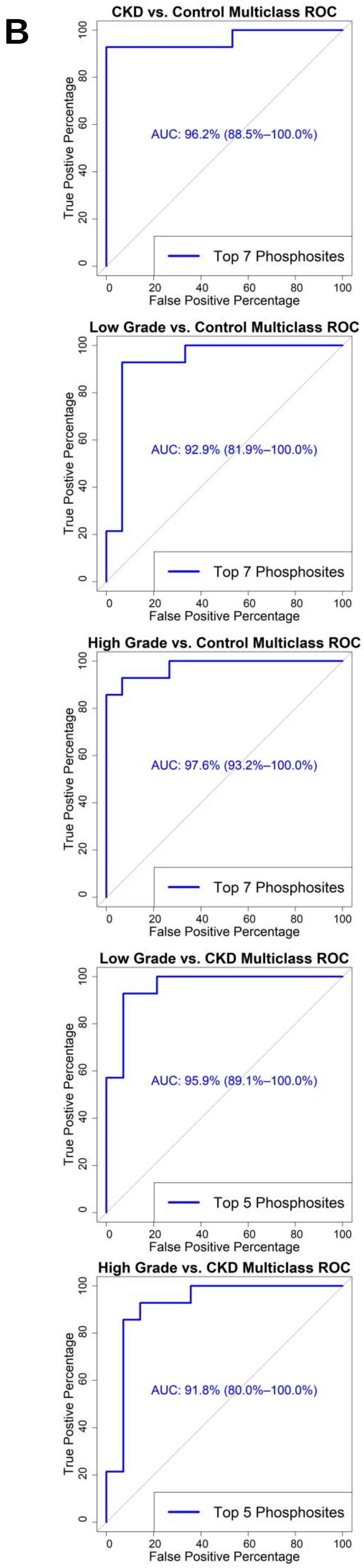

Supplementary Figure 5. Upregulated phosphosite feature selections and multiclass ROC analyses. A) The variable importance plots (VIPs) for the top 15 phosphosites. B) The receiver operating characteristic (ROC) curve for either the top 5 or 7 phosphosites obtained from the VIP as indicated in the figure legends, along with the area under the ROC curve (AUC) score. The 95% confidence intervals were also included for each AUC score.

Supplementary Figure 6

A

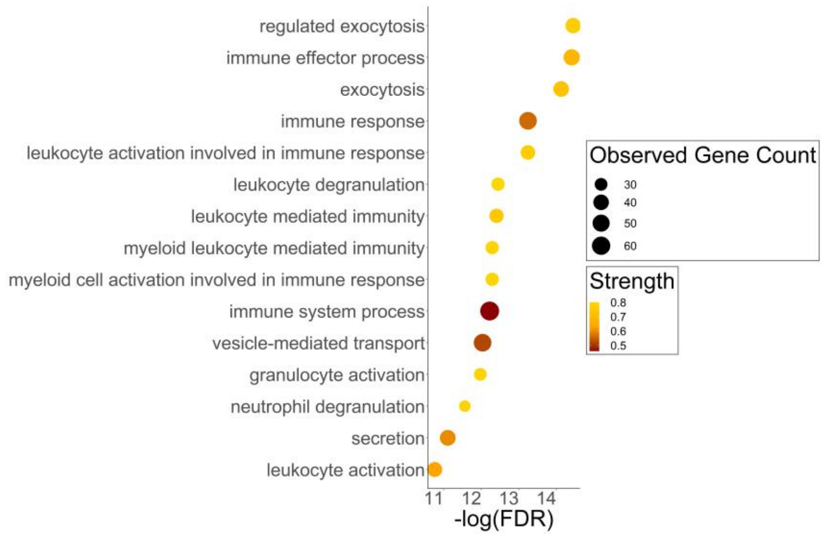

B

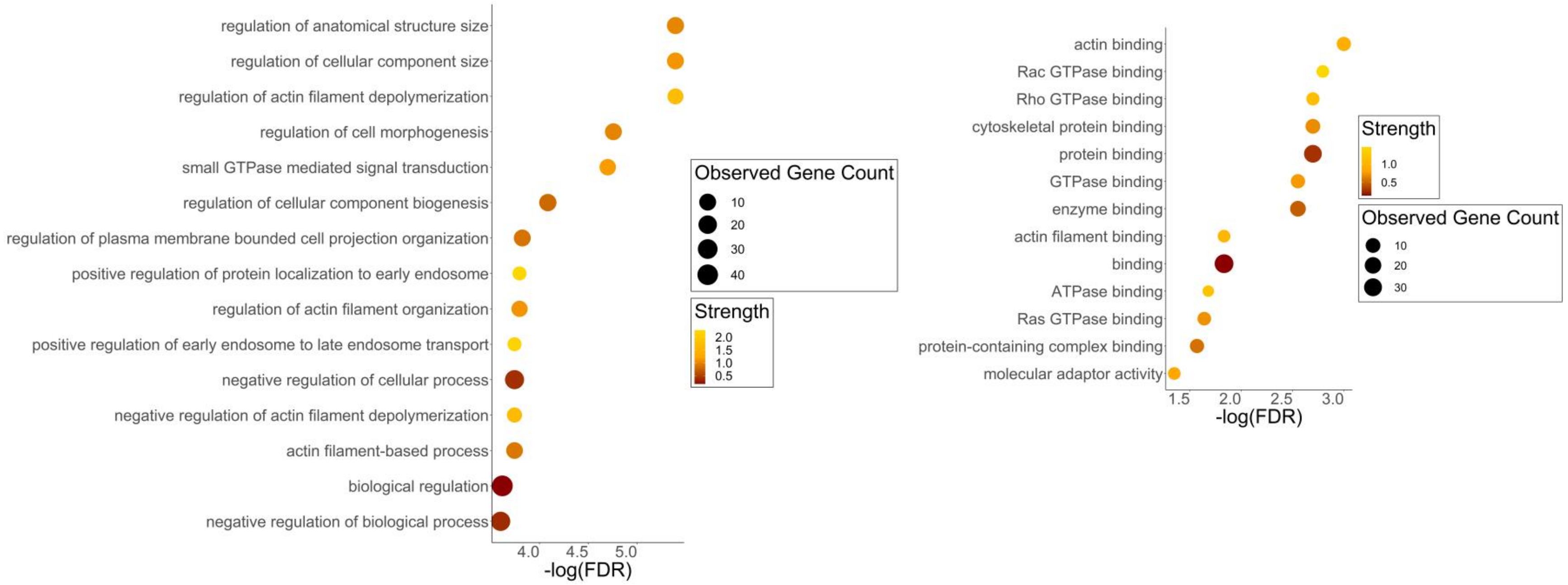

C

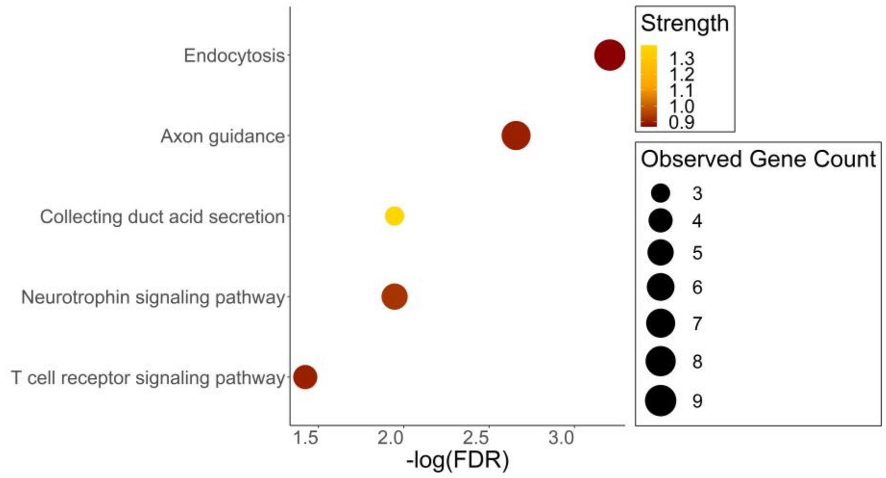

Supplementary Figure 6. Enriched biological process gene ontology analyses of up-regulated phosphosites. The upregulated biological processes for A) CKD compared to control and B) clear cell high-grade compared to control (left) with the upregulated molecular functions for clear cell high-grade compared to control (right). C) The upregulated KEGG for clear cell low-grade compared to CKD. The analyses were carried out with the String database.

Supplementary Figure 7

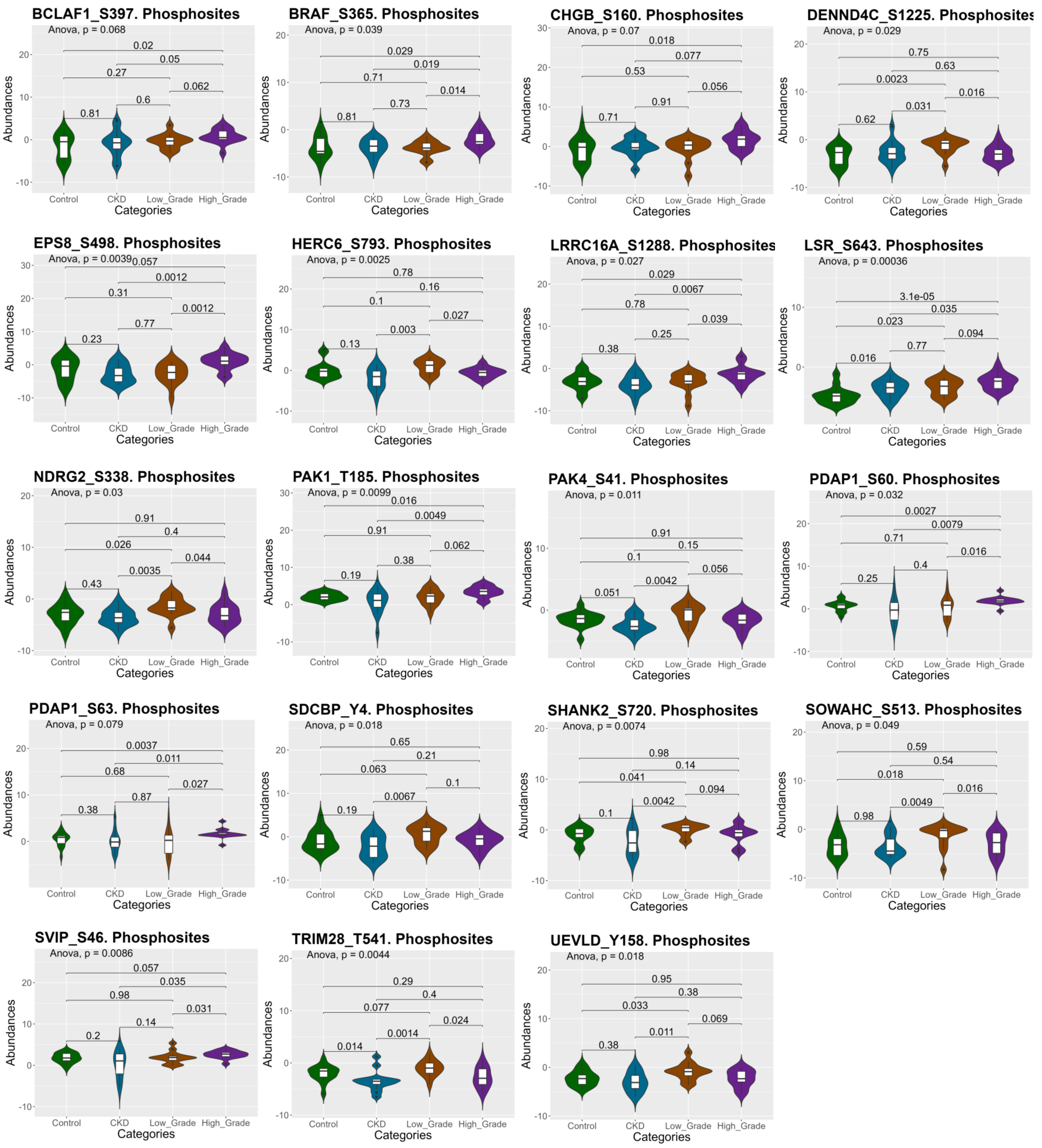

Supplementary Figure 7. Up-regulated phosphosites. The most differentially expressed phosphosites in the low-grade and high-grade clear cell RCC samples as visualized in the heatmap in **Figure 5A** (p-value < 0.1 when low-grade was compared to HC, CKD, or high-grade and when high-grade was compared to HC, CKD, or low-grade, calculated using the unpaired two-samples Wilcoxon test).
